# Supplementary figures and images for: Single-cell RNA sequencing reveals dysregulation of spinal cord cell types in a severe spinal muscular atrophy mouse model
Source: PLoS Genet. 2022 Sep 8;18(9):e1010392. doi: 10.1371/journal.pgen.1010392 (PMC9488758; doi:10.1371/journal.pgen.1010392)

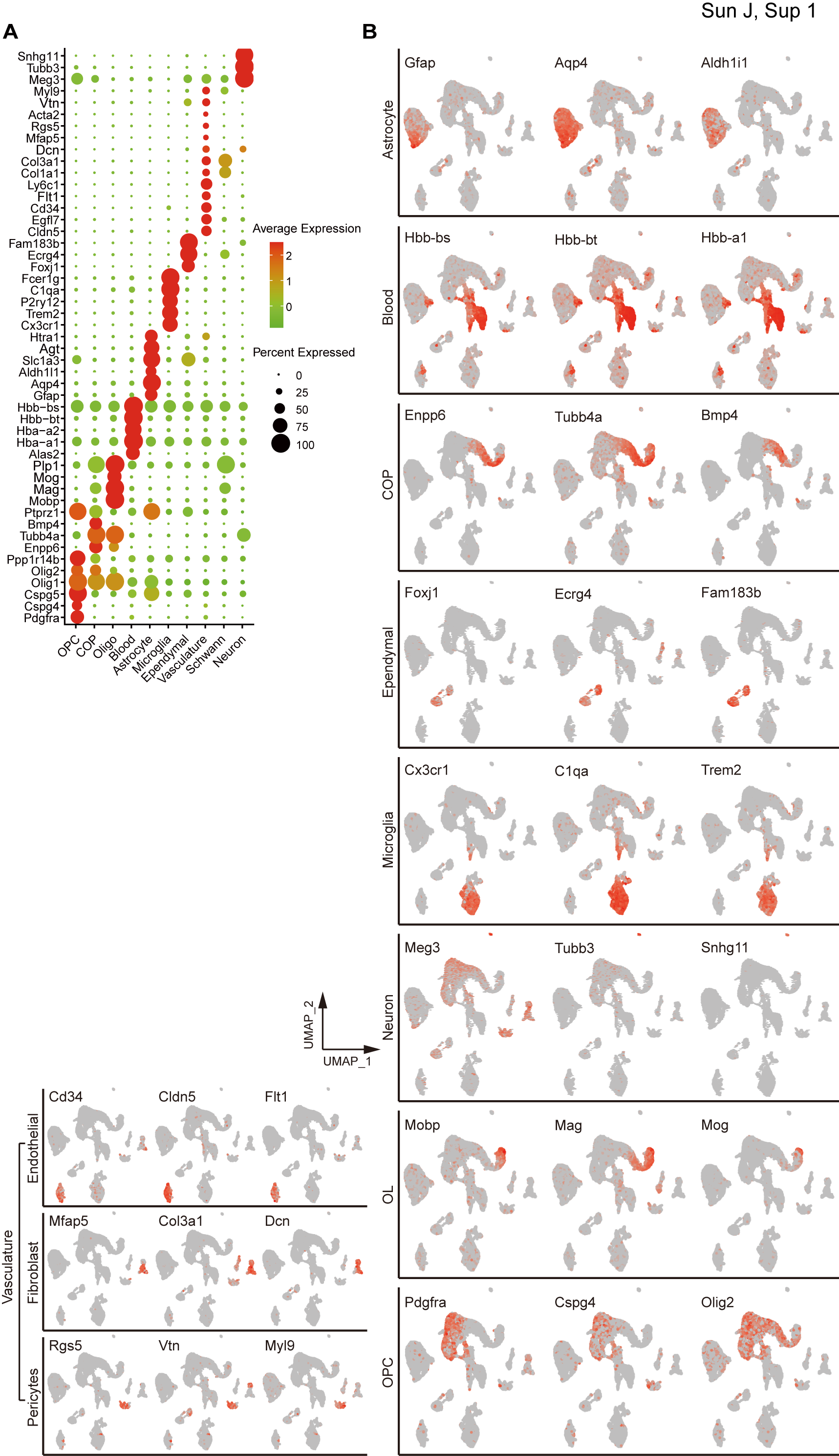

Supplement: S1 Fig — (A) The average expression of each marker gene in different cell types; the darker the red, the higher the expression. (B) UMAP visualization shows the expression of marker genes in different cell types; the darker the red, the higher the expression. (TIF) [file pgen.1010392.s001.tif]

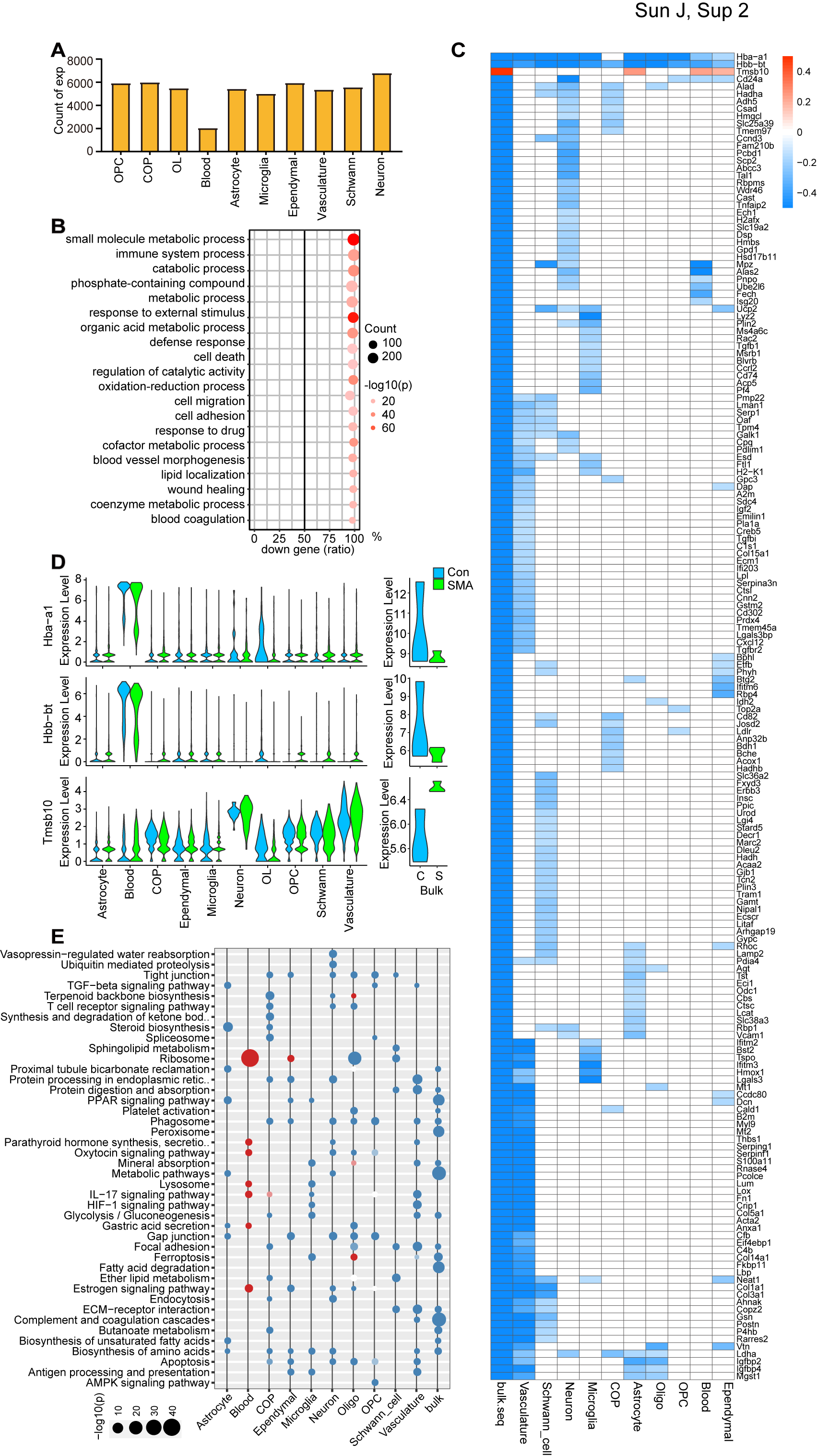

Supplement: S2 Fig — (A) The number of genes detected through scRNA-seq in each cell type. (B) GO analysis of DEGs in bulk-seq. (C) The cell-type-specific DEGs and bulk-seq DEGs were intersected, and the heatmap shows the expression changes in the intersected genes in different cell types and bulk-seq. (D) Violin plots of Hba-a1, Hbb-bt, and Tmsb10 expression in different cell types and bulk-seq. (E) KEGG pathway analysis of cell-type–specific DEGs and DEGs by bulk-seq; red indicates upregulated pathways, and blue indicates downregulated pathways. (TIF) [file pgen.1010392.s002.tif]

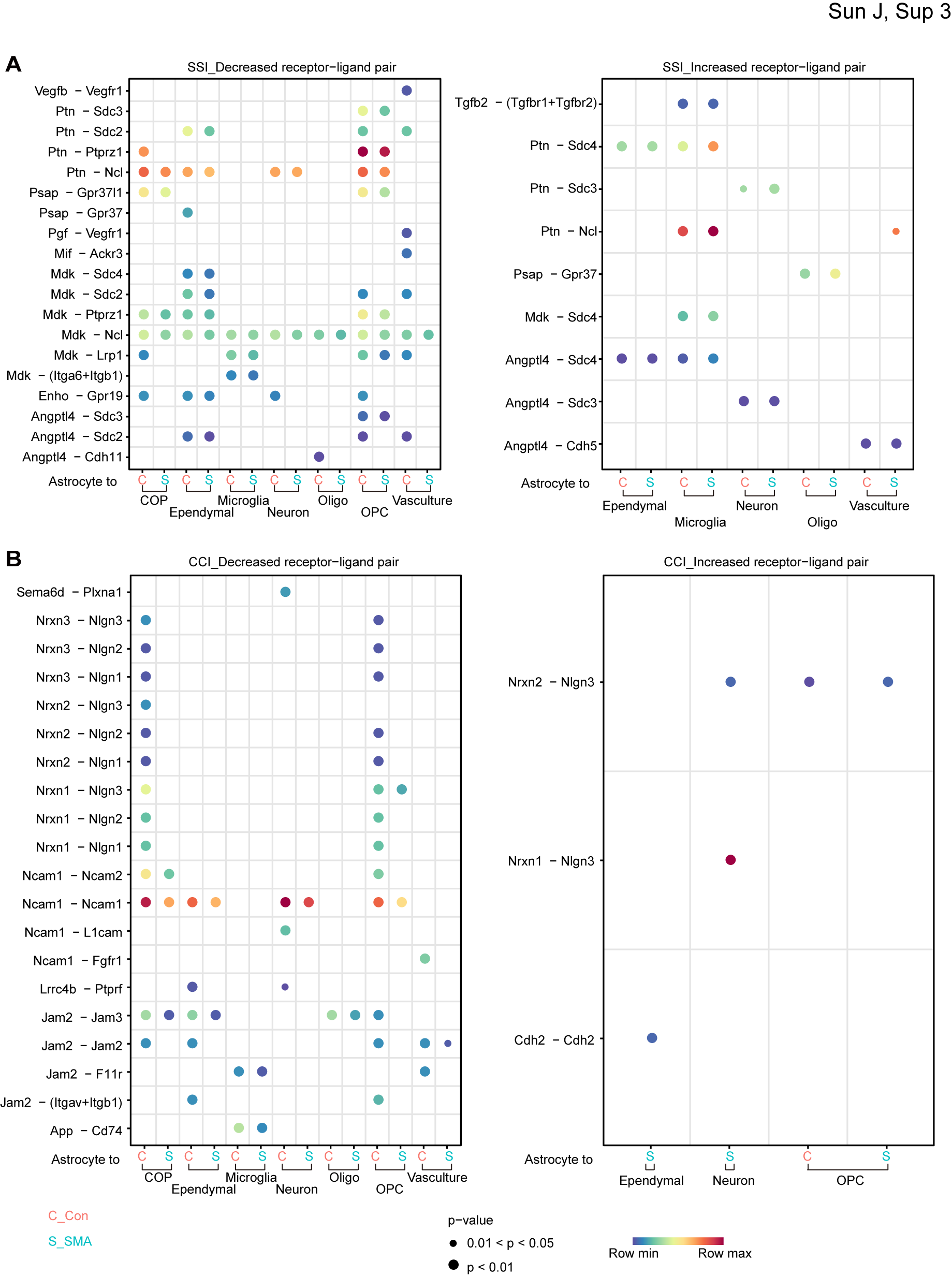

Supplement: S3 Fig — (A) SSI-based receptor-ligand pairings. (B) CCI-based receptor-ligand pairings. (TIF) [file pgen.1010392.s003.tif]

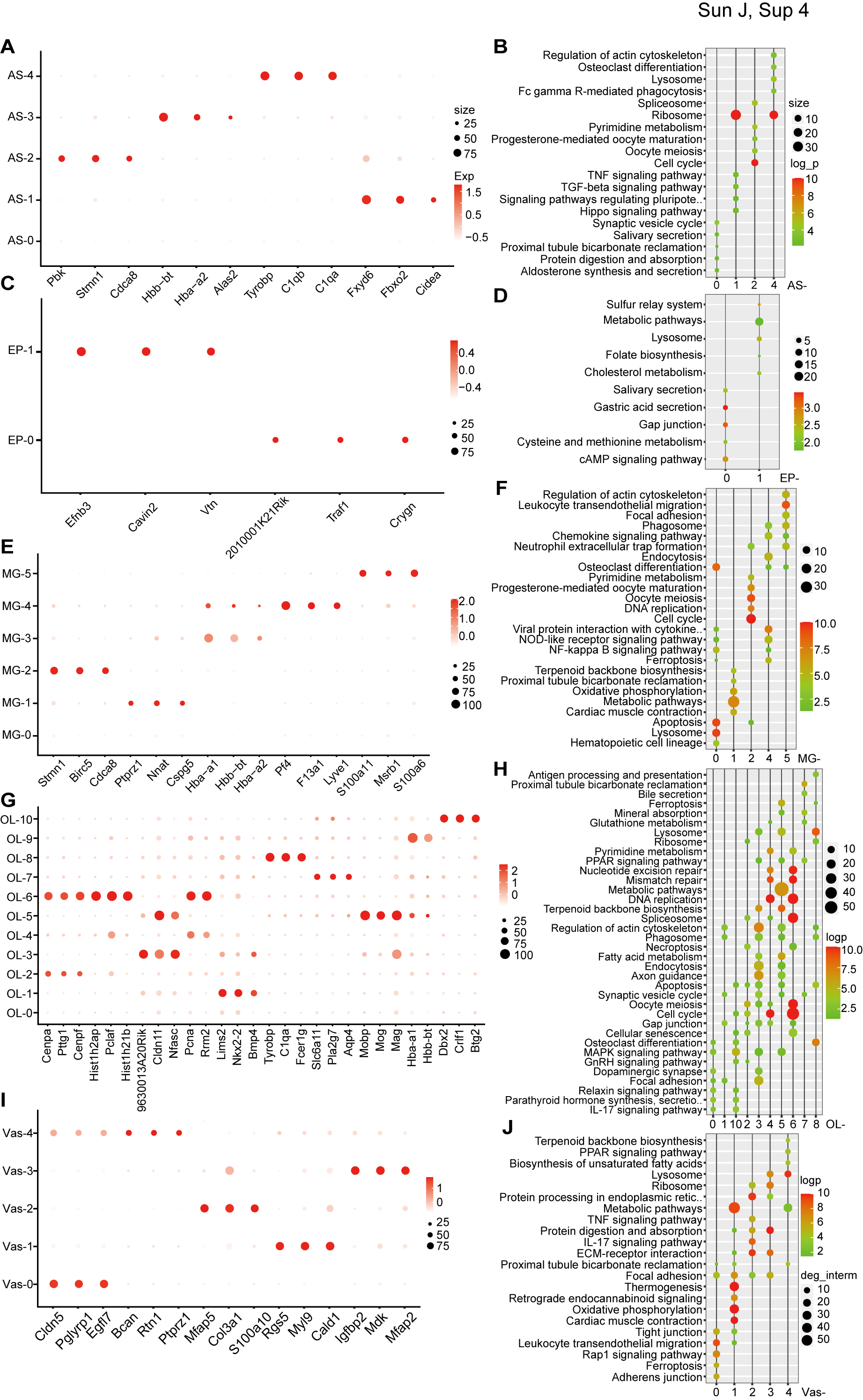

Supplement: S4 Fig — The average expression of markers of each cell subtype of SMA, the darker the red, the higher the expression; A, C, E, G, and I represent astrocytes, ependymal cells, microglia, OL lineages, and vasculature, respectively. KEGG analysis of marker genes for each cell subtype; B, D, F, H, and J represent astrocytes, ependymal cells, microglia, OL lineages, and vasculature, respectively. (TIF) [file pgen.1010392.s004.tif]

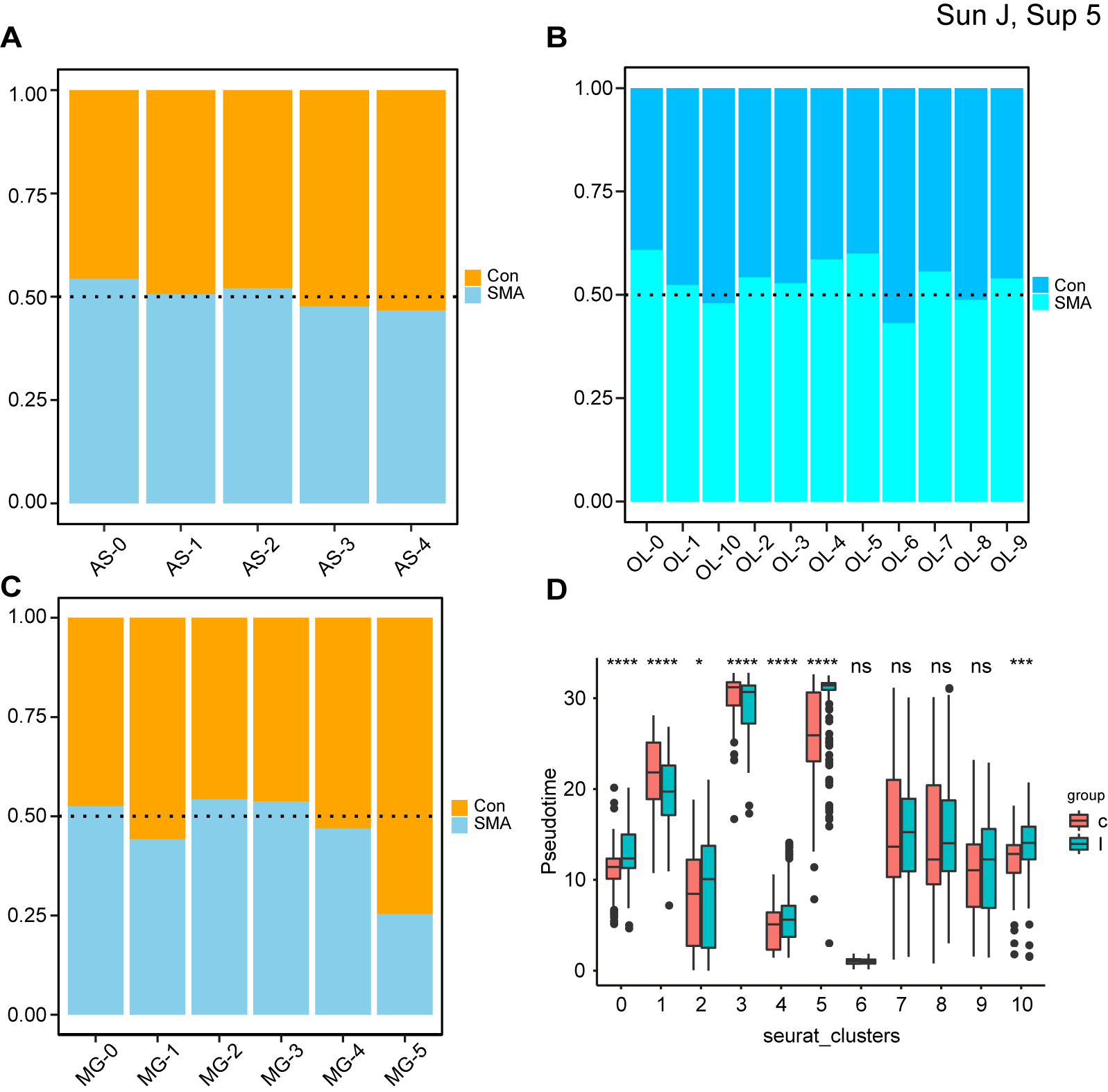

Supplement: S5 Fig — (A–C) Comparison of cell numbers of various subtypes of astrocyte, microglia, and OL lineage in SMA and control mice. (D) Pseudo time analysis of individual cell subtypes in OL lineages. The x-axis represents the cell subtype. The value of the y-axis represents the order of time. The smaller the value, the stronger the characteristics of the progenitor cells. The larger the value, the more mature (later) the cell is. p-values were calculated through the t-test, ns: p > 0.05, *: p < 0.05, ***: p < 0.001, ****: p < 0.0001. (TIF) [file pgen.1010392.s005.tif]

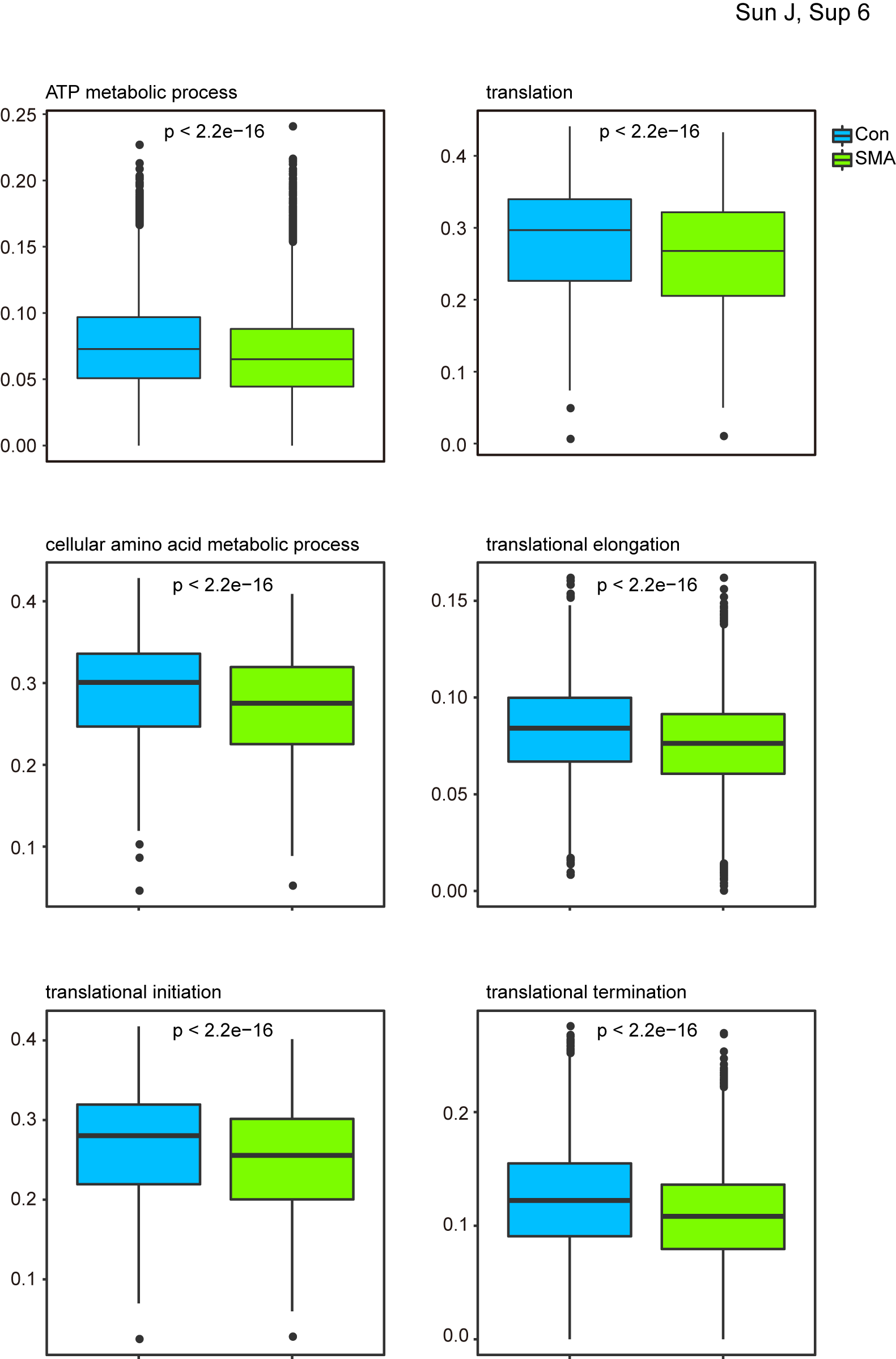

Supplement: S6 Fig — p-values were calculated by t-test. (TIF) [file pgen.1010392.s006.tif]
